# Evolution expresses the second law of thermodynamics in action

**DOI:** 10.1098/rsif.2026.0430

**Published:** 2026-10-07

**Authors:** Arto Annila, Keith Baverstock

**Affiliations:** ^1^ Department of Physics, University of Helsinki, Helsinki, Finland; ^2^ Environmental Science, University of Eastern Finland – Kuopio Campus, Kuopio, Finland

**Keywords:** evolution, natural selection, non-equilibrium thermodynamics, statistical physics, allometric scaling, multiplicative processes, path dependence, complex systems

## Abstract

Evolution through natural selection has long been considered unique to biological systems, yet similar patterns emerge from physical, chemical, technological, economic and social processes. Skewed distributions, often near-lognormal, and sigmoidal courses, often with a power-law central region, recur across scales and scopes, making no distinction between living and non-living. This ubiquity suggests that evolution, in all its manifestations, expresses a universal principle. The second law of thermodynamics, derived from the statistical physics of open systems, explains the recurrent patterns: flows of energy *naturally select* the means and mechanisms that bring about balance in the least time. As a sequence of changes, evolution is destined for the free-energy minimum, equivalent to the entropy maximum; yet its paths remain profoundly unpredictable, even chaotic, as the process alters its own course by consuming its driving forces. By integrating all these interdependencies under the atomistic axiom, the equation of evolution becomes a holistic formulation. Unlike additive genetic models, thermodynamics naturally accounts for multiplicative dynamics and allometric scaling observed in proliferation, adaptation, speciation, cooperation, competition and self-organization, as well as in disintegration, degeneration and extinction. In this way, thermodynamics finally provides the long-sought unified theory from microscopic constituents to macroscopic phenomena.

## 1. Introduction

Evolution by natural selection, as proposed in 1859, traces lineages from the fossil record to the present-day diversity of species. Yet Darwin's tenet does not explain why elemental constituents organize themselves into active agents, nor has physics clarified why matter evolves into functional far-from-equilibrium systems or grounded their perceived purposefulness in fundamental principles.

This theoretical gap is reflected in the gene-centred paradigm's failure to account for biological complexity, with direct implications for clinical practice. For instance, genome-wide association studies (GWAS) [1] capture only a fraction of the expected 50–80% heritability for behavioural traits, and single nucleotide polymorphisms (SNPs) correlate weakly with common diseases arising through multiple pathways, except in rare monogenic disorders where mutations disable irreducible pathways [2–5]. Similarly, gene knock-outs, knock-downs and knock-ins often yield unexpected outcomes that defy Mendelian logic [6,7].

As demonstrated by the Long-Term Evolution Experiment (LTEE), mutations accumulate linearly while fitness increases sigmoidally, contradicting Fisher's theorem that the rate of fitness increase equals the additive genetic variance in fitness [8–10]. Assuming unbiased and statistically independent allelic contributions, the additive genetic variance cannot reproduce the skewed fitness distributions observed in nature [11]. Furthermore, fitness itself cannot be a mere function of genotypic variation, as minimized and native cells exhibit similar adaptive capacities [12].

Epistasis with diminishing returns combined with clonal interference [13] also fails to account for the observed power-law regime of fitness trajectories [14]. In striving to align with the data, evolutionary genetics has adopted concepts such as rugged adaptive landscapes, heterosis, penetrance, expressivity, pleiotropy, genetic drift, epistasis and epigenetics; all remain in active use, though far from well understood [15].

Taken together, the evidence is indisputable: genes do not drive natural processes. Instead, natural processes draw on genes [16] as on everything else in response to forces or ‘evolutionary pressure’. Thus, evolution should be formulated in fundamental, not merely hereditary, terms to explain skewed distributions, path-dependent sigmoid trajectories and multiplicative scaling laws—patterns universal to both animate and inanimate [17–29].

Unknown to most, a holistic evolutionary theory was emerging through statistical physics soon after *On the Origin of Species*. Maxwell's velocity distribution, Boltzmann's energy distribution and Gibbs' chemical equilibria formalized steady-state populations of particles, much like the specialized populations of cells within a mature tissue and populations of species in a climax ecosystem [30–33]. However, these early formulations defined stationary states as if systems were isolated from their invariant surroundings, with total energy and particle numbers held constant.

Subsequently, the system was opened to fluxes under energy conservation, reconceiving organisms as dissipative structures, yet as if far from equilibrium [34,35], rather than considering them in dynamic balance with their high-energy environments. As a result, evolution was long misinterpreted as selecting organisms that maximize entropy export to maintain internal order, incorrectly equated with low entropy. Only recently has evolution towards thermodynamic balance been derived from the principle of least action in its original Maupertuis' open form [36], confirming it as a universal expression of the second law in action [37–39].

This breakthrough in statistical physics affirms that life emerged from matter, not inexplicably, but as a response to the influx of energy from the hot Sun and the efflux to the cold space [40–42]: energy flows themselves *naturally select* the means and mechanisms that consume free energy in the least time. Molecules assemble, cells differentiate, organisms develop and species radiate as all processes across the natural hierarchy tend towards *a free-energy minimum*—that is, *thermodynamic balance* with their respective *evolving surroundings*, a dynamic steady attractor state [43,44]—rather than towards *thermodynamic equilibrium of invariant surroundings* [30–32].

When the surroundings sustain fluxes without appreciable change, the time-translation symmetry [45] is an excellent approximation. However, fluxes do alter the surroundings along with the systems; invariance violates the conservation of quanta and leads theorizing astray. In the irreversibly expanding universe, energy is not conserved: a photon's energy decreases as its period increases. Evolution unfolds with time.

Here, we derive evolution as a natural process from first principles, expressing it as an equation of motion that spans from a primordial molecular pool to present-day species diversity. This thermodynamic formalism explains the entire spectrum of phenomena rather than merely modelling ubiquitous patterns: skewed distributions—from metabolite concentrations to species abundances; sigmoidal trajectories—from single-gene transcription to continental colonization; scaling laws—from body mass to ecosystem biomass and universal standards, such as amino acid chirality and the genetic code.

## 2. Evolution from the first principles

According to thermodynamics, not only do gas atoms, through collisions, and compounds, through reactions, evolve towards balance, but everything, through all kinds of transformations, does as well. By contrast with a reductionist approach, this holistic account maintains a rigorous inventory of every constituent, counting interactions as explicit constituents of the system.

As ancient philosophers proposed and modern physicists proved, both particles and fields are composed of basic building blocks—*atomos* (Greek) or *quantum* (Latin). Each quantum of action measures Planck's constant h=Et with conjugate variables of energy E and time t [46]. As in annihilation and pair production, the conservation of quanta holds because transformations redistribute quanta among particles and the surrounding fields rather than creating or destroying them, unlike the field-theoretic virtual photons. Consequently, all changes are alike: quanta redistribute by consuming free energy, levelling off imbalances. The equation for this irreversible evolution towards ever more probable states is derived by expressing the probability of a state in terms of its assembly.

### 2.1. The probability of a state

Consider a compound, indexed by j, in a primordial pool—for example, an adenine molecule assembled from precursors, such as hydrogen cyanide, ammonia and water. The probability of its existence is the mathematical product,

(2.1) $$p_{j}=\prod_{k}N_{k}\exp[(-\Delta G_{jk}+i\Delta Q_{jk})/k_{B}T],$$

of its constituent k in numbers $N_{k}$, modulated by an exponential factor, determined by the difference in energy $-\Delta G_{jk}$ and the photon energy $i\Delta Q_{jk}$, required to bridge the gap between the compound and its constituents, with both energies normalized by the average energy of the system $k_{B}T$ (figure 1). The prefix i distinguishes the energy carried by the photons from the energy bound in the compounds, ensuring that a state change occurs only through the absorption or emission of a photon [38,47].

Crucially, the product form $\prod_{k}$ ensures that if any of the k constituents were absent altogether, then also $p_{j}=0$. For example, an adenine molecule could not exist if any of its substrates—hydrogen cyanide, ammonia, water or ultraviolet light—were missing altogether from a prebiotic reactor [48], Darwin's ‘warm little pond’. The same accounting by quanta applies to collisional excitation, deforming the electron shell's quantized structure.

**Figure 1.**
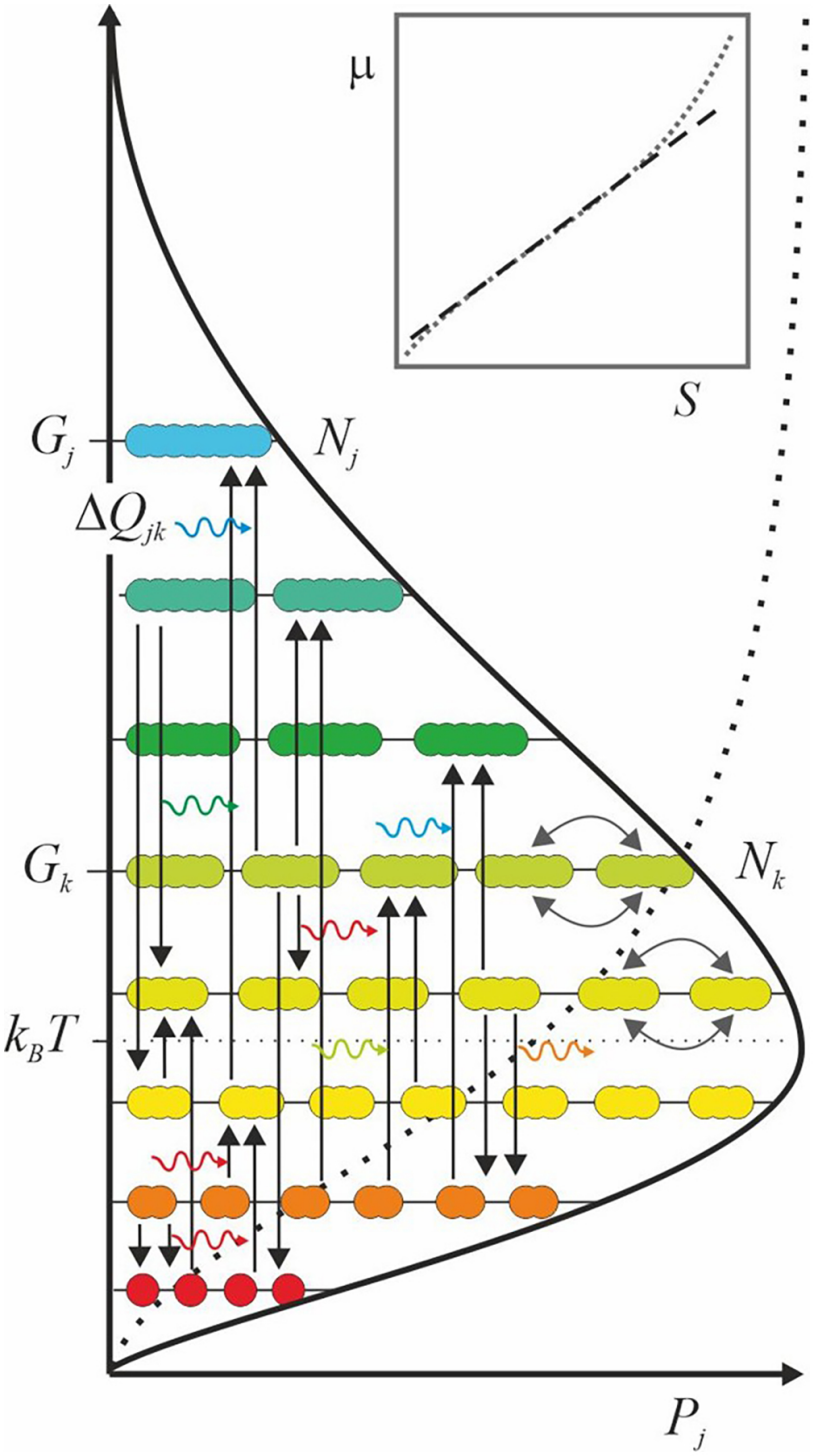
The energy-level diagram depicts a system of compounds assembled from common constituents. Compounds in numbers, $N_{k}$, having the same energy, $G_{k}$, are on the same level. Transformations (vertical arrows) from k ingredients to j products couple light quanta (wavy arrows) carrying energy, $\Delta Q_{jk}$, from the surroundings. Conversely, when the surroundings are low in energy, the system evolves to lower-energy states through reverse transformations, thereby dissipating quanta. Exchange (bow arrows) causes no change in the average energy of the system, $k_{B}T$. The cumulative probability distribution curve (dotted line) is a sigmoid. When its logarithm, entropy, S, is plotted as a function of (chemical) potential energy, μ, it mainly follows a power law, i.e. a straight line on the logarithm–logarithm scale (inset).

The multiplicative probability (equation (2.1)) manifests itself, for example, in ATP synthesis, which fails to start if any required factor—ADP, phosphate, proton motive force, photon energy or redox energy—is absent. Similarly, transcription fails to start if any required factor—polymerase, nucleotides, cofactors or regulatory proteins—is missing. Thus, the formalism explains why biological functions are vulnerable to the loss of any vital component, yet robust to fluctuations in the number of any one component when all are present.

The multiplicative form $P_{j}=(p_{j}{)}^{N_{j}}/N_{j}!$ of a population ensures that if any of the j compounds in numbers $N_{j}$ is missing, then $P_{j}=0$. The factor $1/N_{j}!$ accounts for the indistinguishability of identical compounds; novelty does not arise from permutations of entities but from the influx or efflux of quanta into and from the surroundings [49].

Finally, the total P for a system with all $N_{j}$,

(2.2) $$P=\prod_{j=1}P_{j}=\prod_{j=1}{\left[\prod_{k=1}N_{k}\exp\left(\frac{-\Delta G_{jk}+i\Delta Q_{jk}}{k_{B}T}\right)\right]}^{N_{j}}/N_{j}!\;\;,$$

counts all energies bound in the populations of ingredients k and products j, including the energy of photons $i\Delta Q_{jk}$ in jk transformations that bridge the energy gaps $-\Delta G_{jk}$ relative to the average energy of the system $k_{B}T$. For example, photon influx drives photosynthesis in chloroplasts, each of which contains myriad j and k components. Similarly, thermal efflux is required for mitochondrial catabolism.

Numerous quantized changes, each perturbing $k_{B}T$ only slightly, limit to continuous compounding, $f(x)=e^{x}$. This function, self-similar under change $f^{\prime }(x)=e^{x}$, appears in the canonical distributions of statistical mechanics: Boltzmann statistics for populations on energy levels, Fermi–Dirac statistics for those further constrained by the exclusion principle and Bose–Einstein statistics for the spectrum of photons between these levels. Accordingly, the emission and absorption spectra reflect the statistical distributions of the components in a given system.

The main result, the probability (equation (2.2)), quantifies the state of any system, since all systems consist of quanta—from atoms and molecules to organelles, cells, organisms, communities and ecosystems. Thus, evolution across all scales towards increasingly probable states can be expressed by the time derivative dP/dt.

### 2.2. The equation of state

For historical reasons, ln⁡P, as an additive measure, is multiplied by the Boltzmann constant $k_{B}$ to define entropy:

(2.3) $$S=k_{B}\ln P=k_{B}\sum_{j=1}\ln P_{j}\approx \frac{1}{T}\sum_{j,k=1}N_{j}(-\Delta \mu_{jk}+i\Delta Q_{jk}+k_{B}T),$$

where the approximation follows from Stirling's formula $\ln N_{j}!\approx N_{j}\ln N_{j}-N_{j}$.

No single universally accepted definition of entropy exists for open systems far from equilibrium [50]. The entropy in equation (2.3) is based on the atomistic axiom and sums over states that differ in energy rather than over energetically indistinguishable microstates, as in Boltzmann's $k_{B}\ln W$.

The total energy TS accounts for the energy bound in populations $\sum N_{j}k_{B}T$ and for the energy free in chemical potential differences $\Delta \mu_{jk}=\mu_{j}-\mu_{k}$ and in endothermic influxes or exothermic effluxes $i\Delta Q_{jk}$ between the system and its surroundings. The potentials $\mu_{k}=k_{B}T\ln N_{k}+G_{k}$ and $\mu_{j}=k_{B}T\ln N_{j}+G_{j}$ quantify all kinds of populations. Consequently, S here is not synonymous with disorder or decoherence in phase space, nor is it an assembly index [51]. As transformations through the absorption and emission of quanta alter paths, including those of atomic and molecular orbitals, the associated phase relations change and coherence disperses. The lost coherence reappears as system–environment correlations [38,52–54].

### 2.3. The equation of evolution

Differentiating the state equation (equation (2.3)) with respect to time using the chain rule yields

(2.4) $$T\frac{dS}{dt}=T\sum_{j=1}\frac{dS}{dN_{j}}\frac{dN_{j}}{dt}=\sum_{j,k=1}\frac{dN_{j}}{dt}\left(-\Delta \mu_{jk}+i\Delta Q_{jk}\right),$$

totalling the changes along the energy gradients. The average energy $k_{B}T$ sums the energies of all constituents; it is not differentiated. The summation remains open to new populations emerging from quanta of free energy (exergy); in turn, populations decline as they lose quanta, eventually to extinction. For instance, in microbial evolution experiments, fitness increases when new metabolic pathways access new sources of free energy. Accordingly, when nutrients are exhausted, population growth stalls because the free-energy term vanishes.

Equation (2.4) expresses, in every detail, the evolution Darwin portrayed with its fullness of life. Notably, evolution cannot be predicted because, by detailed balance [55],

(2.5) $$\frac{dN_{j}}{dt}=\frac{1}{k_{B}T}\sum_{k}\sigma_{jk}\left(-\Delta \mu_{jk}+i\Delta Q_{jk}\right)=-\sum_{k}\frac{dN_{k}}{dt},$$

population changes $dN_{j}/dt$, proportional to mechanism-dependent factors $\sigma_{jk}>0$, are inseparable from $N_{j}$ contained in $\mu_{j}=k_{B}T\ln N_{j}+G_{j}$ of the endothermic or exothermic free energy terms $-\Delta \mu_{jk}+i\Delta Q_{jk}$; outcomes derive from all states along the path, not just the initial one [56]. Such context dependence shows up in gene knock-outs: removing an enzyme blocks a pathway, while driving forces reroute flows in other ways.

The surroundings, as sources and sinks of fluxes, coevolve with the system, in line with Maupertuis' principle of least action, which projects non-deterministic trajectories whose final states remain open from the outset. The inexact differential $d\;{\;}^{-}Q$ captures the path dependence in the thermodynamic identity $dS=d\;{\;}^{-}Q/T$.

Path dependence is evident in embryogenesis, morphogenesis and phylogenesis [57–60], driven by numerous and varying forces, less so in idealized physics, where motion is attributed to a single invariant force. Thus, neither deterministic models, which prescribe unique trajectories from given initial conditions, nor stochastic ones, such as Gaussian, Markovian or Poisson processes, nor chaotic models sensitive to initial conditions accurately capture natural processes by treating the driving forces as independent of the changes they cause.

Although fundamentally inaccurate, determinism approximates non-determinism when changes consume only a minute fraction of the dominant force, as with a falling stone. Conversely, indeterminism can approximate non-determinism when energy differences between alternatives are negligible, as with tossing a coin. However, such approximations rarely hold in complex systems, where forces and changes are inextricably coupled.

According to equations (2.4) and (2.5), quanta flow along the steepest gradients in free energy. Any deviation from these geodesics would entail a spontaneous non-physical creation of force carriers, violating the conservation of quanta—echoing the spontaneous non-physical ‘vital force’ posited in vitalism.

It is worth highlighting that natural processes do not face energy barriers to overcome—a common misconception arising from the kinetic interpretation of the law of mass action [39]. Instead, kinetics (equation (2.5)) is fully consistent with thermodynamics (equation (2.4)), as rates are proportional to free energy, temperature and mechanisms ($\sigma_{jk}$). A reaction proceeds when the energy of the reactants—the sum of potential, kinetic and absorbed quanta—exceeds that of the products and accelerates, for example, when a catalyst widens bottlenecks for both forward and reverse flows rather than reshaping the energy landscape.

The mechanisms themselves are systems that have evolved to function optimally. Evolution both diverges and converges as mechanisms reorganize to consume free energy in the least time, across scales—from enzymes at the molecular level and organelles at the cellular level to specialized traits at the level of organisms and cultural or technological practices at the level of societies.

Free energy decreases and entropy increases without exception: dS/dt≥0 follows from inserting equation (2.5) into equation (2.4) and squaring the free-energy terms, orthogonal in the jk basis. As a result, natural processes become irreversible as forces for return are depleted and mechanisms displaced. This plays out as pioneer species with generalist functions giving way to successors that harvest more free energy across specific niches through specialized functions. Thus, the trophic hierarchy reemerges, and adaptive radiation recurs after disturbances or catastrophes. The height of hierarchy is limited by the breadth of its base, as reflected in the trophic pyramids of the species–area relationship.

Within the ever-evolving universe, a natural process approaches—though never attains—the dynamic free-energy minimum of TdS/dt=0. Its homeostatic nature derives from Lyapunov stability: deviation $\delta N_{j}$ away from the steady state $N_{j}=\prod_{k}N_{k}\exp[(-\Delta G_{jk}+i\Delta Q_{jk})/k_{B}T]$ generates balance-restoring forces, rendering TdS/dt>0 and $Td^{2}S/dt^{2}<0$. Demonstrating Le Chatelier's principle, populations respond to changing conditions, eventually all the way to extinction. Since the thermodynamic aim is not to maximize diversity but to minimize free energy, monocultures persist and expand even in natural habitats [61].

Rather than narrowly gauging fitness by reproductive success or other one-sided criteria, the second law captures the full capacity and complexity of free-energy consumption down to a single quantum (equation (2.4)). The energy flows themselves *naturally select* functions, including standards such as genetic code, common metabolites and amino acid handedness, as the least-time paths towards balance [62,63]. Obsolete functions are lost, as seen in cave-dwelling fish evolving blindness, ground-dwelling birds developing vestigial wings or nutrient-limited bacteria switching codon usage to minimize the cost of expression. This quest for efficiency also drives evolutionary arms races.

Thermodynamics makes clear that the energy gradient between the solar source and the cosmic sink powers the biosphere [29,40,41,64], just as the free energy in fossil fuels drives matter into firms, industries, economies and societies [65–67]. However, if these fluxes are left out of the equations, life appears as a far-from-equilibrium structure dissipating into cold space rather than as processes that consume free energy. If free energy is not accounted for, theorizing becomes incoherent and an artificial abiotic–biotic divide emerges, obscuring the universality of patterns in the data.

The principle of least-time free-energy consumption parallels the principle of maximum entropy production (MEP). However, when MEP is formulated using the information entropy $-k_{B}\sum_{i}p_{i}\log p_{i}$, it describes the exchange of phase configurations (microstates) under a fixed total probability $\sum_{i}p_{i}=1$, rather than evolution towards balance. In turn, Prigogine's near-equilibrium principle of minimum entropy production minimizes dissipation [35,68]. Neither maximum nor minimum entropy production accounts for natural processes that consume free energy in the least time (equation (2.4)).

## 3. The universal patterns

As noted long ago [69], sigmoidal evolutionary trajectories (equation (2.5)), manifested as punctuated equilibria [70], with long periods of stasis alternating with rapid bursts of diversification, stand in contrast to gradualism. When free energy, $-\Delta \mu_{jk}+i\Delta Q_{jk}$, becomes abundantly available, primal mechanisms, $\sigma_{jk}$, limit the rate of consumption:

(3.1) $$\begin{array}{rl} \frac{d}{dt}\frac{1}{k_{B}T}\sum_{k=1}\left(-\Delta \mu_{jk}+i\Delta Q_{jk}\right) & =\frac{dN_{j}}{dt}\frac{d}{dN_{j}}\frac{1}{k_{B}T}\sum_{k=1}\left(-\Delta \mu_{jk}+i\Delta Q_{jk}\right)\approx \sum_{k=1}\sigma_{jk}\\ \Rightarrow \;\frac{dN_{j}}{N_{j}} & =\sum_{k=1}\sigma_{jk}dt\Rightarrow N_{j}(t)=N_{j}(0)\exp(\sum_{k=1}\sigma_{jk}t), \end{array}$$

where $d\mu_{j}/dN_{j}=d(G_{j}+k_{B}T\ln N_{j})/dN_{j}=k_{B}T/N_{j}$, while $\mu_{k}$ and $\Delta Q_{jk}$ depend on $N_{j}$ only by stoichiometry. The initial growth bursts nearly exponentially as primitive mechanics consume a seemingly endless supply. As this early change in free energy is approximately constant, a simple separation of variables yields an exponential rise. Similarly, $N_{j}(t)$ levels off almost exponentially near balance as mature machinery consumes the final narrow margins at peak efficiency, at an approximately constant rate. In economics, the pattern emerges as technology matures: early productivity rises quickly, but subsequent gains come with diminishing returns, eventually stalling entirely.

During the intermediate phase, the multiplicative assembly of entities yields a power-law course, as populations $N_{j}=\prod_{k}[N_{k}\exp(-\Delta G_{jk}+i\Delta Q_{jk})]=\alpha_{j}N_{1}^{j}$ assemble from the basic building blocks $N_{1}$ consuming free energy in numerous forms $\alpha_{j}=\prod_{mn}\exp[(-\Delta \mu_{mn}+i\Delta Q_{mn})/k_{B}T]$. Thus, the change

(3.2) $$\frac{dN_{j}}{dt}=\frac{dN_{j}}{dN_{1}}\frac{dN_{1}}{dt}=j\alpha_{j}N_{1}^{j-1}\frac{dN_{1}}{dt}=j\frac{N_{j}}{N_{1}}\frac{dN_{1}}{dt}\Rightarrow \frac{dN_{j}}{N_{j}}=j\frac{dN_{1}}{N_{1}}$$

integrates to a power law $\ln N_{j}=j\ln N_{1}+$ const.

The well-documented allometric laws, for example, relating metabolic rate to body mass and species number to area, thus follow from the product form of P, which, when transformed via ln⁡P, yields straight lines on log–log plots, often spanning many orders of magnitude and appearing scale-free [22–29]. For example, power-law correlations between SNPs and traits, common diseases or drug responses reflect numerous interdependencies rather than being reducible to simple one-to-one causal relationships.

The complete sigmoidal form spanned by equations (3.1) and (3.2) recurs across processes. For example, bacterial colony growth accelerates when nutrients are abundant, decelerates as resources become limiting and plateaus at carrying capacity—matching the early-exponential, mid-power-law and late-exponential phases derived from the thermodynamic principle (equations (2.4) and (2.5)). Consistently, fitness trajectories in LTEE follow a sigmoidal curve: early mutations unlock large free-energy gains, and later mutations yield diminishing returns as the system approaches balance. Developmental processes such as embryogenesis, wound healing and tumour growth follow the same pattern because they, too, are driven by the consumption of free energy.

Rapid population dynamics can be modelled by adding the feedback term $-\beta N_{j}$ to equation (3.1):

(3.3) $$\frac{dN_{j}}{N_{j}}\approx (\sum_{k=1}\sigma_{jk}-\beta N_{j})dt\;\Rightarrow N_{j}(t)=N_{j}(t_{o})(1+\sum_{k=1}\sigma_{jk}\Delta t-\beta \Delta tN_{j}(t_{o})),$$

which relates the population, $N_{j}(t_{o})$, at a time, $t_{o}$, to the population, $N_{j}(t)$, at a later time, $t=t_{o}+\Delta t$ [71]. When a population devours resources faster than they can be replenished, oscillations arise from the mismatch between consumption and resumption [39,72]. However, trajectories may even become chaotic when the statistical approximation $|-\;\Delta \mu_{jk}+i\Delta Q_{jk}|/k_{B}T\ll 1$ no longer holds owing to major perturbations such as extreme weather and severe pests.

In thermodynamics, all kinds of differences, not merely mutations, give rise to variation. Accordingly, a distribution with variation, j±n, about the mean density in energy, $\phi_{j}=N_{j}\exp(G_{j}/k_{B}T)$, expressed in terms of the elemental density, $\phi_{1}=N_{1}\exp(G_{1}/k_{B}T)$ (cf. equation (2.2)),

(3.4) $$\ln\phi_{j-n\ldots j+n}=\ln\phi_{j}+\sum_{n}n\ln\phi_{1}$$

holds for n≪j and is then nearly lognormal by the central limit theorem, with tails extending beyond those of a normal distribution. The near-lognormal distribution emerges from multiplicative causal processes, whereas the normal distribution arises from additive random processes.

Once to-and-fro fluxes become non-negligible, the small-step assumption $|-\Delta \mu_{jk}+i\Delta Q_{jk}|/k_{B}T\ll 1$ breaks down, and increments no longer average out independently but correlate. Then, the tail fattens from the exponential decay (equation (3.1)) into the polynomial regime, where free energy scales with population (equation (3.2)). For example, earthquake magnitudes follow a power law truncated at the maximum fault size; wealth follows a power law truncated by finite resources; species abundances follow a power law truncated by finite habitat size. Also, spirals are energetically optimal shapes in polar coordinates [39]: pine-cone scales and nautilus shells approximate lognormal distributions [73,74]; galaxies exhibit a power law at the faint end with an exponential decay at the bright end [75] and cyclone cloud areas follow a fat-tailed distribution [76].

Surprises lurk in the long tails of natural distributions. For example, effects and countereffects cascade throughout the ecosystem when an invading species goes viral. Mathematically speaking, the system revolves around the introduced singularity. The eigenvalues of the monodromy matrix, L≡dln⁡P, defined by equation (2.4), relate to orbital stabilities and frequencies. Once the invader is fully integrated, the steady-state L becomes skew-symmetric. Perturbations may still trigger oscillations, but no longer explosive instability.

The thermodynamic formalism also clarifies why genetic variation alone cannot predict phenotypes: the variation in free-energy consumption, not the variation in alleles, determines evolutionary outcomes. For example, SNP–trait associations follow power-law distributions because each SNP contributes multiplicatively to the free-energy landscape through metabolic, structural or regulatory effects. This explains why GWAS signals are diffuse, why epistasis is pervasive and why minimal cells retain adaptive capacity despite lacking many genes: what matters is the system's ability to consume free energy, not the specific genetic configuration.

Equations (2.4) and (2.5), deriving from the atomistic axiom of indivisible quanta, make sense of natural processes in coherent, causal and contingent terms, while their reduced statistical forms—lognormal, logistic and power-law-like curves—fit empirical data without explaining why the data take those forms. Coarse-grained models, scale-free networks [77], evolutionary game theory [78] and the Lotka–Volterra equations reproduce data patterns without explaining why they arise [79–81]. The master equations of stochastic processes, such as Markovian, branching and diffusion models, capture changes without explaining why they occur. Capturing a pattern is not capturing a cause. A cause answers why by naming the driving force; a mechanism answers how by mapping the flow of quanta.

## 4. Discussion

‘An exact determination of the laws of heredity will probably work more change in man's outlook on the world, and in his power over nature, than any other advance in natural knowledge that can be clearly foreseen’, declares William Bateson in the opening of *Mendel's Principles of Heredity: A Defence* (1902). Speaking to horticulturists, he goes on to say, ‘We want to know the whole truth of the matter; we want to know the physical basis, the inward and essential nature, “the causes,” as they are sometimes called, of heredity: but we want also to know the laws which the outward and visible phenomena obey’. However, contrary to Bateson's assertion, Mendelism has not stood the test of time as a law of nature; instead, the flexible framework of the Modern Synthesis, subsuming Darwin's theory of natural selection and Mendelian genetics, has emerged as data have accumulated.

Then again, rather than focusing on mechanisms, genetic or otherwise, evolution by natural selection can be regarded as a law of nature [82], one that explains patterns that recur across scales. From molecules to organisms, from communities to civilizations, and from stars to galaxies, evolution unfolds along sigmoidal trajectories, yielding skewed distributions. This universality derived from atomism cannot be confined to Mendelian genetics, but must be recognized as the expression of a universal law in action, giving rise to complex characteristics with a broad spectrum, such as intelligence [83,84].

As we have shown, the ubiquitous patterns in the data derive from the second law of thermodynamics in action: energy flows *naturally select* means and mechanisms to reach balance in the least time. Darwin's finches diversified to tap into different energy sources. Across continents, anteaters evolved convergently to consume a widespread energy supply. Predators and prey, parasites and hosts and plants and herbivores evolved to gain or regain access to free energy, while other organisms secured it through symbiosis. As past paradigm shifts show [85], a more comprehensive theory not only extends but also elevates understanding; the principles of physics transcend the conceptual scope of biology. As abiogenesis implies, thermodynamics makes no distinction between inanimate and animate, or natural and artificial, or microscopic and cosmic.

However, classical physics, with its deterministic trajectories, cannot account for path-dependent, non-deterministic evolution [56], nor does the statistical determinism or indeterminism of modern physics explain changes through fluxes of quanta. As the surroundings coevolve with the system, the boundary conditions shift—the integration limits of the least-action principle—rendering trajectories inherently open. Accordingly, thermodynamics derived from the statistical physics of open quantum systems clarifies emergence and quantum phenomena by tracking the fluxes of quanta [49,54].

By the atomistic axiom, information is embodied in physical substance [86–88], and hence subject to the second law, unlike its formal definitions by Shannon and Jaynes, which depend on how the observer groups microstates into macrostates and what the observer presumes about those macrostates. Because information is physical, genes do not force but facilitate change, just as books do not cause but catalyse change [10]. Mechanisms are not causes but consequences, emerging for channelling flows [89]. Thus, it is illogical to regard the products of evolution, such as genes, as prerequisites, and even less as prime movers [90]. A gene is a material cause that codes cellular functions, which serve as efficient causes subordinate to the final cause of consuming free energy. Every open system, from a cell to the biosphere, evolves by consuming free energy towards such a local minimum—an asymptotic ideal nested within the universe's own grand asymptotic approach to heat death.

Thermodynamics makes clear that history unfolds along the paths taken. Ergo, the future remains open wherever free energy is available, including what is at our disposal. Fossil fuels power processes only for a moment, whereas sunlight drives them across epochs; its immense force is best balanced by keeping the Earth green with forests, resilient through species diversity.

## Data Availability

This article has no additional data.
